# Response to the “Glasgow Prognostic Score as A Marker of Mortality After TAVI”

**DOI:** 10.21470/1678-9741-2021-0577

**Published:** 2022

**Authors:** Ipek Buber

**Affiliations:** 1 Cardiology Department, Pamukkale University, Denizli, Kinikli, Turkey. E-mail: dr.ipekbuber@gmail.com

Dear Editor,

I have read the article entitled “Glasgow Prognostic Score as a Marker of Mortality after TAVI”, by Abacioglu et al. and published in the BJCVS, with great interest^[1]^. The investigators reported that the Glasgow prognostic score (GPS) is an easy, noninvasive laboratory test which may be used as a predictive biomarker for outcomes in patients undergoing transcatheter aortic valve implantation (TAVI).

Aortic stenosis is one of the most common valve diseases in the worldwide population^[2]^, and the increase in the elderly population increases the frequency of TAVI, which is a well-established alternative to surgical valve replacement in patients at high surgical risk^[3]^. Conventional scoring systems, including logistic European System for Cardiac Operative Risk Evaluation (EuroSCORE), EuroSCORE II, and the Society of Thoracic Surgeons risk score, are used to determine the surgical risk in patients undergoing TAVI^[4]^. However, these traditional risk scoring systems are sometimes inadequate to predict periprocedural and long-term mortality in patients undergoing TAVI. GPS is an inflammation-based prognostic score evaluated using the elevation of C-reactive protein and decrease in albumin concentration^[5]^. It is usually used to assess the risk in cancer patients undergoing surgery^[6]^, and this score predicts the outcomes for idiopathic pulmonary fibrosis^[7]^, systemic lupus erythematosus^[8]^, and inflammatory bowel diseases^[9]^.

In this context, assessment of the GPS value, which is an easy and noninvasive tool, can be beneficial to determine the prognosis of patients undergoing TAVI because there is a requirement for new scores that will provide prognostic information in TAVI patients.
